# A Model for the Magnetoimpedance Effect in Non-Symmetric Nanostructured Multilayered Films with Ferrogel Coverings

**DOI:** 10.3390/s21155151

**Published:** 2021-07-29

**Authors:** Nikita A. Buznikov, Galina V. Kurlyandskaya

**Affiliations:** 1 Scientific and Research Institute of Natural Gases and Gas Technologies–Gazprom VNIIGAZ, Vidnoye, Razvilka, 142717 Moscow, Russia; n_buznikov@mail.ru; 2 Department of Electricity and Electronics, Basque Country University UPV/EHU, 48940 Leioa, Spain; 3 Department of Magnetism and Magnetic Nanomaterials, Institute of Natural Sciences and Mathematics, Ural Federal University, 620002 Ekaterinburg, Russia

**Keywords:** magnetic multilayers, magnetoimpedance, modeling, magnetic sensors, magnetic biosensors, magnetizable nanoparticles, ferrogels

## Abstract

Magnetoimpedance (MI) biosensors for the detection of in-tissue incorporated magnetic nanoparticles are a subject of special interest. The possibility of the detection of the ferrogel samples mimicking the natural tissues with nanoparticles was proven previously for symmetric MI thin-film multilayers. In this work, in order to describe the MI effect in non-symmetric multilayered elements covered by ferrogel layer we propose an electromagnetic model based on a solution of the 4Maxwell equations. The approach is based on the previous calculations of the distribution of electromagnetic fields in the non-symmetric multilayers further developed for the case of the ferrogel covering. The role of the asymmetry of the film on the MI response of the multilayer–ferrogel structure is analyzed in the details. The MI field and frequency dependences, the concentration dependences of the MI for fixed frequencies and the frequency dependence of the concentration sensitivities are obtained for the detection process by both symmetric and non-symmetric MI structures.

## 1. Introduction

Demands for fast development of small biomedical devices have increased the interest in magnetic biosensors. They are compact analytical devices with magnetic physico-chemical transducers for the evaluation of the concentration of components of interest [1,2]. In general, magnetic sensors can be divided into two groups: magnetic sensors for label-free detection processes [3,4] and magnetic devices for detection of magnetic labels [1,2,5,6]. Although label-free magnetic detector prototypes were of special interest recently [7,8], a major part of the studies is related to the possibility to detect magnetizable nanoparticle concentrations [9,10]. There are different solutions for the detection: in “in vitro” experimental models [1,10,11,12], nanoparticles inside living cells [13,14], in continuous flow in medical devices or in blood flow [15,16], as a part of the implants or embedded into a natural tissue or artificial composites mimicking a natural tissue [5,17,18,19]. Many types of magnetic field sensors were tested in a simple “laboratory” device configuration just to ensure the proof of the concept. A recent overview might be useful for advanced reading on this subject [20]. The improved levels of sensitivity now available with respect to the applied magnetic field allow one to propose very new applications of magnetic field sensors which would be unthinkable at lower levels of sensitivity [21,22].

**Figure 1:**
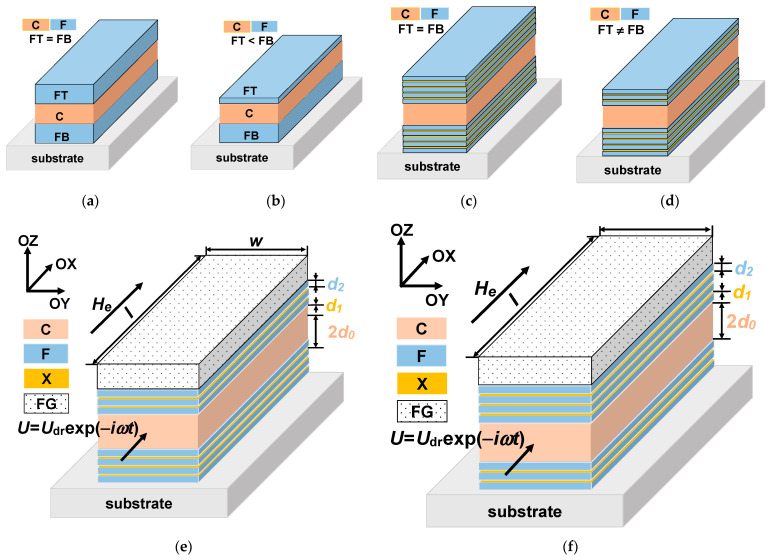
Schematic representation of the MI multilayered structures. Symmetric structures of “sandwich” type with equal thicknesses of ferromagnetic and conductive layers (**a**). Non-symmetric structure of “sandwich” type with different thicknesses of ferromagnetic top and bottom layers (**b**). Symmetric MI structure with equal thicknesses of top and bottom identical multilayers consisting of ferromagnetic soft magnetic sub-layers separated by non-ferromagnetic spacers and conductive central layer (**c**). Non-symmetric MI structures with different thicknesses of top and bottom multilayers consisting of ferromagnetic soft magnetic sub-layers separated by non-ferromagnetic spacers and conductive central layer (**d**). Studied non-symmetric multilayered films: the multilayer with ferrogel placed onto the surface of thin layer (**e**) and onto the surface of thick layer (**f**).

Magnetoimpedance (MI) is one of the effects ensuring very high sensitivity with respect to magnetic field and detection of fields of the order of up to 10^–8^ Oe. The MI phenomenon consists in the change of the total impedance of a ferromagnetic conductor in the presence of an external magnetic field [23]. The interest in MI is related to the design of small magnetic-field detectors for different application areas–from non-destructive testing and vehicle control to the magnetic detection of the signals closely related to the living system functionality and magnetic marker detection [9,19,24]. The magnetoimpedance phenomenon was previously observed and studied in different soft ferromagnets obtained by rapid quenching, electrodeposition, magnetron sputtering and other methods [25]. However, from the point of view of the compatibility with existing semiconductor electronics and in view of specially requested packaging of sensor array and miniaturization, multilayered film-based MI elements with the total thickness of magnetic layers are one of the most suitable magnetic materials for magnetoimpedance applications [26]. The MI effect was predicted to reach the highest value, theoretically described and experimentally tested for three-layered structures in MI “sandwich” configuration (Figure 1a) for which two ferromagnetic layers (F) of the equal thickness are separated by the conductive non-ferromagnetic central layer (C) of the same thickness as the ferromagnetic layers [27,28]. In this case, when the thickness of the top (FT) and the bottom (FB) ferromagnetic layers is the same, the structure is called “symmetric” [29]. As the theory predicted the highest MI value for symmetric structures, the non-symmetric structures (with FT ≠ FB, Figure 1b) were not experimentally studied.

In the case of permalloy thin films, the need of nanostructuring, i.e., substitution of thick ferromagnetic FeNi layers by the multilayered structures of [F/X]_m_ (Figure 1c) was rather carefully discussed in the literature [30,31]. This step is necessary due to the existence of the transition into a “transcritical” state [31,32]. Non-symmetric multilayered structures (Figure 1d) were studied experimentally and theoretically [26,29]. Non-symmetric MI structures with open magnetic flux can be obtained by sputtering deposition of top and bottom ferromagnetic layers with different thickness [5,26]. However, there are no theoretical studies for the case of magnetic nanoparticles (MNPs) detection by a magnetic biosensor with non-symmetric MI elements (Figure 1d).

In the course of the development of MI thin-film-based prototypes for the detection of MNPs embedded in natural tissue an intermediate solution was proposed. Synthetic composites consisting of a polymer matrix with embedded MNPs–ferrogels substituted for the biological tissues at the first stage of the development of the biosensor [5,17]. MNPs in ferrogel are dispersed swollen in water forming an elastic cross-linked polymeric network. The mobility of MNPs in a ferrogel depends on two parameters: the diameter of the MNPs and the distance between adjacent crosslinks of the gel network. Ferrogels’ properties in many senses can mimic the main properties of biological samples, being similar to the cytoskeleton. Their structure in the simplest way can be described as a polymer network with electric charges localized on macromolecule filaments of the network and free counterions. The last are dispersed inside the polymeric network in the liquid phase [17,33]. Although ferrogels-covering detection by the MI symmetric structures was demonstrated experimentally and a satisfactory model was proposed [5,34], the non-symmetric structures for such detection were still not analyzed.

In this work, we propose a new model in order to describe the MI effect in non-symmetric multilayered elements covered by a ferrogel layer. The theoretical approach is based on the previous calculations of the distribution of electromagnetic fields in non-symmetric multilayered films further developed for the case of the ferrogel covering. The influence of the asymmetry of the film on the MI response of the multilayer–ferrogel structure is analyzed.

## 2. Model

### 2.1. Field Disrtibution and MI in Multilayer–Ferrogel Structure

The studied [F/X]_m_/F/C/[F/X]_n_/F multilayered structure is a rectangular MI element with transverse effective magnetic anisotropy. The induced magnetic anisotropy is formed due to application of the technological magnetic field during multilayer deposition. The MI element consists of a highly conductive non-ferromagnetic central layer C of a thickness 2*d*_0_ and two external multilayers: ferromagnetic top and bottom parts. The external multilayers contain soft magnetic sub-layers F of thickness *d*_2_ separated by non-ferromagnetic spacers X of thickness *d*_1_. The corresponding conductivities of the materials C, X and F are *σ*_0_, *σ*_1_ and *σ*_2_. The multilayered element length and width are *l* and *w*, respectively. The film structure is non-symmetric, that is, the top external multilayer is either thicker than the bottom one, *m* > *n*, either thinner than the bottom multilayer, *m* < *n*. The layer of ferrogel with the thickness of *d*_3_ is placed on the top surface of the film structure (Figure 1e,f). The driving voltage is used to feed the element: *U* = *U*_dr_exp (−*iωt*) (*t* is the time *ω* is the angular frequency of the electromagnetic filed and *i* is the imaginary unit).

The driving voltage is applied to the MI element (Figure 1e) in the geometry of longitudinal MI. the external magnetic field *H_e_* is parallel to the long side of the element in the flowing current direction. We assume the dependence of the electromagnetic fields on the coordinate perpendicular to the film plane (*x*-coordinate) only. Such an assumption is possible due to the fact that the length of the multilayer and its width are much higher than the thickness of the multi-layered structure. That is it the one-dimensional approximation is used. The Maxwell equations can be solved and the amplitudes of the longitudinal electric *e_j_* and the transverse magnetic *h_j_* fields in the sub-layers can be written as follows [29]:

(1) $$e_{j}=(cp_{k}/4\pi \sigma_{k})[A_{j}\cosh(p_{k}x)+B_{j}\sinh(p_{k}x)],$$

(2) $$h_{j}=A_{j}\sinh(p_{k}x)+B_{j}\cosh(p_{k}x).$$

Here *j* = 1,…2 (*m* + *n* + 1) + 1 is the sub-layer number; subscript *k* = 0, 1 and 2 corresponds to the central conductive layer, spacer and magnetic sub-layer, respectively. *A_j_* and *B_j_* are the constants; *p_k_* = (1 − *i*)/*δ_k_*; *δ_k_* = *c*/(2*πωσ_k_μ_k_*)^1/2^; *c* is the speed of light in vacuum; *σ_k_* and *μ_k_* are the conductivity and the transverse permeability values for the material *k*. For the non-ferromagmagnetic central layer and spacers we assume *μ*_0_ = *μ*_1_ = 1.

The distribution of the fields within ferrogel layer can be expressed as follows [35]:

(3) $$e_{g}=[A_{g}\cosh(p_{3}x)+B_{g}\sinh(p_{3}x)]/\varepsilon^{1/2},$$

(4) $$h_{g}=A_{g}\sinh(p_{3}x)+B_{g}\cosh(p_{3}x).$$

Here *e_g_* and *h_g_* are the amplitudes of the electric and magnetic field in the ferrogel; *A_g_* and *B_g_* are the constants; *ε* is the permittivity of the ferrogel and *p*_3_ = −*iωε*^1/2^/*c*.

In order to describe the distribution of the field outside the structure consisting of a MI multilayered element and a ferrogel the approximate solution for the vector potential in the previously obtained general form was used [36,37]. The corresponding field amplitudes in the particular geometry for the external regions can be be expressed as follows:

(5) $$e_{\mathrm{ext},q}=C_{q}\frac{i\omega l}{2cw}\left[\frac{l}{2w}\ln\left(\frac{R+w}{R-w}\right)-\frac{4x}{l}\arctan\left(\frac{wl}{2Rx}\right)+\frac{w}{l}\ln\left(\frac{R+l}{R-l}\right)\right],$$

(6) $$h_{\mathrm{ext},q}=C_{q}(2/w)\arctan(wl/2Rx).$$

Here the subscripts *q* = 1 and *q* = 2 correspond to the bottom and top external region, respectively; *C_q_* are the constants and *R* = (*l*^2^ + *w*^2^ + 4*x*^2^)^1/2^.

To describe completely the distribution of the electromagnetic fields within the studied structure, the constants in Equations (1)–(6) should be found. The continuity conditions for the amplitudes of the electric and magnetic fields at the interfaces between different sub-layers of the film, *j* < 2 (*m* + *n* + 1), can be presented in the form:

(7) $$e_{j}=e_{j+1}\;,\;\;h_{j}=h_{j+1}.$$

Additional restrictions for the values of the field amplitudes are obtained from the excitation condition of the multilayered film. We should take into account that the driving voltage is applied to the multilayer region −*t*_1_ < *x* < *t*_2_, where *t*_1_ = *d*_0_ + *nd*_1_ + (*n* + 1)*d*_2_ and *t*_2_ = *d*_0_ + *md*_1_ + (*m* + 1)*d*_2_. Then, the boundary conditions are given by the following expressions:

(8) $$\begin{array}{l} e_{\mathrm{ext},1}(-t_{1})+U_{\mathrm{dr}}/l=e_{1}(-t_{1})\;,\\ h_{\mathrm{ext},1}(-t_{1})=h_{1}(-t_{1}-d_{3})\;,\\ e_{2(m+n+1)+1}(t_{2})=e_{g}(t_{2})+U_{\mathrm{dr}}/l\;,\\ h_{2(m+n+1)+1}(t_{2})=h_{g}(t_{2})\;,\\ e_{g}(t_{2}+d_{3})=e_{\mathrm{ext},2}(t_{2}+d_{3})\;,\\ h_{g}(t_{2}+d_{3})=h_{\mathrm{ext},2}(t_{2}+d_{3})\;. \end{array}$$

Equations (7) and (8) allow one to find all constants in Equations (1)–(6). After that, the impedance *Z* of the multilayered structure can be obtained as a ratio of the applied voltage to the total current flowing through the multilayer [33,34]:

(9) $$Z=U_{\mathrm{dr}}{\left[w\int_{-t_{1}}^{t_{2}}\sigma (x)e(x)dx\right]}^{-1}=\frac{4\pi }{cw}\times \frac{U_{\mathrm{dr}}}{h_{2(m+n+1)+1}(t_{2})-h_{1}(-t_{1})}\;.$$

### 2.2. Static Magnetization Distribution and Transverese Permeability

The field and frequency dependences of the MI response in the multilayered structure are determined by the transverse permeability *μ*_2_ in the soft magnetic sub-layers. At relatively high frequencies, the permeability *μ*_2_ is governed by the magnetization rotation [25]. It is assumed that all magnetic sub-layers have uniaxial in-plane magnetic anisotropy, and the angle *ψ* of deviation of the anisotropy axis from the transverse direction is relatively small. The deviation of the anisotropy axis from the transverse direction in real multilayers is related to the influence of different factors, in particular, the shape anisotropy and local non-uniformities.

The influence of the ferrogel layer on the MI response is related to stray fields induced by MNPs. The stray fields change the magnetization distribution in the soft magnetic sub-layers and affect the transverse permeability *μ*_2_. To describe the influence of the stray fields on the MI effect it is assumed that the ferrogel layer generates a spatially uniform effective field *H_p_* in the multilayer [34,35]. The value of *H_p_* is proportional to the concentration of MNPs in the ferrogel, since it was found that the ferrogel saturation magnetization *M*_sat_ increases linearly with the concentration of MNPs of iron oxide which are biocompatible materials widely used in biomedical applications [34,36,38].

Following the approach developed previously [34], the dependence of the ferrogel magnetization *M_g_* on the external magnetic field is approximated by the following linear function:

(10) $$M_{g}=M_{\mathrm{sat}}(H_{e}-H_{c})/(H_{1}-H_{c}).$$

where *H_c_* is the coercive force of the ferrogel and *H*_1_ is the value of the external magnetic field close saturation, i.e., *M_g_* ≈ *M*_sat_. By the assumption, the effective field *H_p_* is oriented in the opposite direction with respect to the magnetization vector in the layer of ferrogel. To simplify calculations, we assume that the effective stray field *H_p_* is proportional to the ferrogel saturation magnetization *M*_sat_. In addition, we neglect the spatial distribution of the field *H_p_* over the multilayer thickness. Although the approach simplifies the real distribution of the stray fields, it allows one to describe qualitatively the effect of the MNPs on the MI response in the multilayer–ferrogel structure.

The magnetization distribution in the magnetic sub-layers can be calculated by the minimization of the free energy. Because of the above described procedure [34] the following equation for the equilibrium magnetization angle *θ* can be obtained:

(11) $$H_{a}\sin(\theta -\psi )\cos(\theta -\psi )-H_{p}\sin(\theta -\varphi )-H_{e}\cos\theta =0.$$

Here *H_a_* is the anisotropy field in the magnetic sub-layers and *φ* = arcsin(*M_g_*/*M*_sat_). Note that at *H_e_* << *H*_1_, Equation (11) can be simplified since *φ* ≈ 0.

The transverse permeability *μ*_2_ in the soft magnetic sub-layers can be found by means of solution of the linearized Landau–Lifshitz equation [25], which results in the following expression [34]:

(12) $$\mu_{2}=1+\frac{\omega_{m}[\omega_{m}+\omega_{1}-i\kappa \omega ]\sin^{2}\theta }{[\omega_{m}+\omega_{1}-i\kappa \omega ][\omega_{1}-\gamma H_{a}\sin^{2}(\theta -\psi )-i\kappa \omega ]-\omega^{2}}.$$

Here *ω_m_* = 4*πγM*, *M* is the saturation magnetization of the magnetic sub-layers, *γ* is the gyromagnetic constant, *κ* is the Gilbert damping parameter and:

(13) $$\omega_{1}=\gamma [H_{a}\cos^{2}(\theta -\psi )-H_{p}\cos(\theta -\varphi )+H_{e}\sin\theta ].$$

## 3. Results

The proposed model allows one to analyze the MI effect of non-symmetric multilayered elements with a ferrogel covering. The simulations were carried out for multilayered films with a copper central layer, permalloy Fe_20_Ni_80_ magnetic sub-layers with close to zero magnetostriction and titanium spacers.

Further, we use the following parameters of the permalloy sub-layers: the saturation magnetization *M* = 750 G, the conductivity *σ*_2_ = 3·10^16^ s^−1^, the anisotropy field *H_a_* = 6 Oe, the anisotropy axis deviation angle *ψ* = −0.1*π* and the Gilbert damping parameter *κ* = 0.02. It is assumed that the thickness of the copper central layer 2*d*_0_ = 500 nm and the conductivity *σ*_0_ = 5·10^17^ s^−1^. For the titanium spacers, we take *d*_1_ = 3 nm and *σ*_1_=5·10^16^ s^−1^. The length of the multilayers is 1 cm and their width is 0.2 mm. The ferrogel layer thickness is 1 mm, and the permittivity of the ferrogel *ε* = 80.

**Figure 2:**
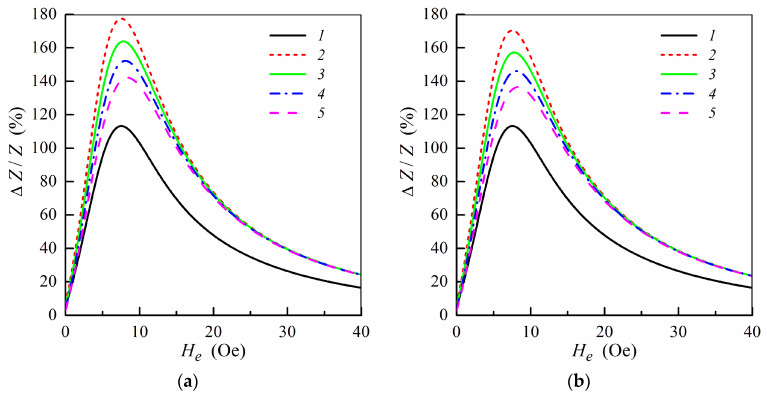
MI ratio Δ*Z*/*Z* as a function of the external field *H_e_* at *f* = *ω*/2*π* = 100 MHz for the multilayered element with ferrogel placed onto the surface of thin layer (*m* = 3, *n* = 4) (**a**) and onto the surface of thick layer (*m* = 4, *n* = 3) (**b**) (see also Figure 1e,f). Curves 1, multilayered film without gel; curves 2, film with pure gel (hydrogel) (*H_p_* = 0); curves 3, *H_p_* = 0.25 Oe; curves 4, *H_p_* = 0.5 Oe; curves 5, *H_p_* = 0.75 Oe. Parameters used for calculations are *l* = 1 cm, *w* = 0.2 mm, 2*d*_0_ = 500 nm, *d*_1_ = 3 nm, *d*_2_ = 100 nm, *M* = 750 G, *H_a_* = 6 Oe, *ψ* = −0.1π, *σ*_0_ = 5 × 10^17^ s^−1^, *σ*_1_ = 5 × 10^16^ s^−1^, *σ*_2_ = 3 × 10^16^ s^−1^, *κ* = 0.02, *d*_3_ = 1 mm, *H_c_* = 6.5 Oe, *H*_1_ = 750 Oe and *ε* = 80.

To describe a relative variation of the impedance we introduce the MI ratio Δ*Z*/*Z*, which is given by the following relation: Δ*Z*/*Z* = [*Z*(*H_e_*) − *Z*(*H*_0_)]/*Z*(*H*_0_), where *H*_0_ = 100 Oe is the external field sufficient for magnetic saturation of the multilayered structure. It was found that for multilayers with the parameters mentioned above, the maximum values of the MI ratio are achieved within the frequency range from 50 to 100 MHz [29]. Figure 2 shows the field dependence of the MI ratio Δ*Z*/*Z* calculated at the frequency *f* = *ω*/2*π* = 100 MHz for the non-symmetric multilayer without ferrogel and multilayer with ferrogel for different values of the effective stray field *H_p_*. The results are presented only for the range of the positive fields, since the MI ratio is symmetric with respect to the sign of the external field. The pure gel is a hydrogel without MNPs, for which stray fields are equal to zero (*H_p_* = 0) [34,35].

When the gel sample is placed onto the surface of the magnetoimpedance element (Figure 1e,f) MI ratio increases due to high permittivity of the gel as it was observed previously for the symmetric structures [33,34]. The effective stray field increases with the concentration of MNPs in the ferrogel due to a growth of the ferrogel saturation magnetization. As a result, the MI ratio decreases with an increase of the stray field *H_p_*_._ However, observed behavior (in the case of non-symmetric MI elements) is differ from the results obtained for corresponding symmetric structures [34,35].

It follows from Figure 2 that the MI ratio Δ*Z*/*Z* is higher when the ferrogel layer is placed onto the surface of the thin layer. The dependence of the MI ratio on the position of the ferrogel layer is related to changes in the distribution of the electromagnetic fields within the multilayer in the presence of the ferrogel. It was demonstrated that the MI ratio is maximal for the symmetric multilayer without ferrogel at not too high frequencies [29]. Thus, the symmetric distribution of the electromagnetic fields within the multilayer is preferable in order to achieve highest values of the MI ratio. In the case when the ferrogel is placed onto the surface of the thin layer, the field distribution within the multilayer becomes more symmetric. On the contrary, when the ferrogel is placed onto the surface of the thick layer, the asymmetry in the field distribution is enhanced, which results in the decrease of the MI ratio (Figure 2).

**Figure 3:**
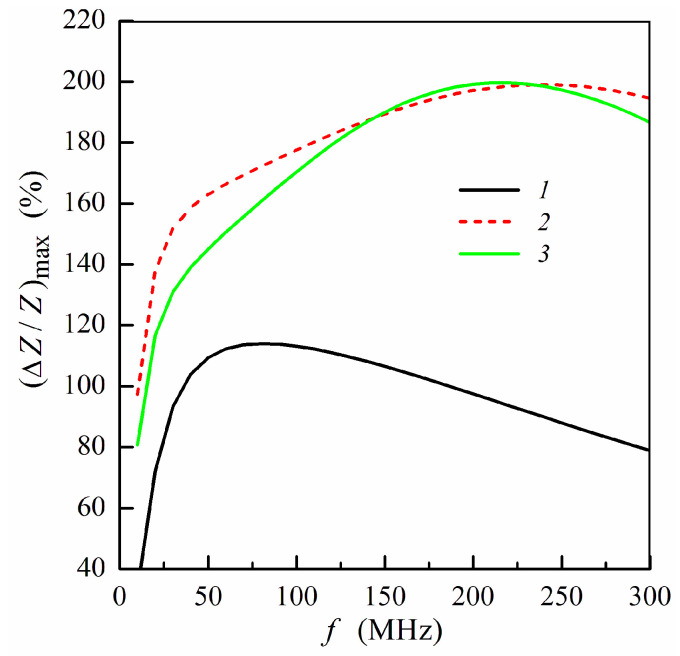
Frequency dependence of maximum MI ratio (Δ*Z*/*Z*)_max_: curve 1, multilayered film without gel; curve 2, multilayer with pure gel (hydrogel) placed onto the surface of thin layer (*m* = 3, *n* = 4); curve 3, multilayer with pure gel placed onto the surface of thick layer (*m* = 4, *n* = 3) (see also Figure 1e,f). Other parameters used for calculations are the same as in Figure 2.

To analyze the effect of the ferrogel layer positioning with respect to the magnetic layers of the non-symmetric structure on the MI response, we use the maximum MI ratio (Δ*Z*/*Z*)_max_, which corresponds to the maximum value of Δ*Z*/*Z* at a fixed frequency. Figure 3 shows the frequency dependence of (Δ*Z*/*Z*)_max_ calculated for the non-symmetric multilayer with different positions of the pure gel (hydrogel) without MNPs. Within the low frequency range, the application of the gel onto the surface of thin layer (Figure 1e) allows one to obtain higher values of (Δ*Z*/*Z*)_max_.

There is a frequency range approximately from 25 to 50 MHz where the difference between MI ratio for two positions under consideration exceeds 10% of (Δ*Z*/*Z*)_max_, which is sizeable difference. At sufficiently high frequencies (above 150 MHz), the values of the maximum MI ratio depend slightly on the gel position: whether it is placed onto the thin or thick multilayer part (Figure 3).

**Figure 4:**
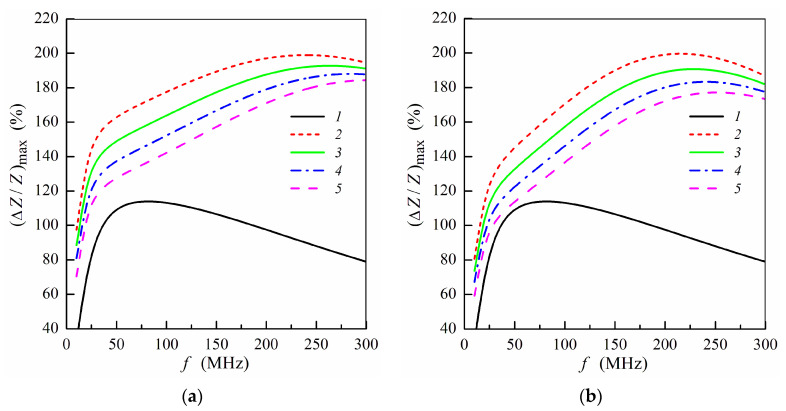
Frequency dependence of maximum MI ratio (Δ*Z*/*Z*)_max_ for the multilayered sensitive element with ferrogel placed onto the surface of thin layer (*m* = 3, *n* = 4) (**a**) or onto the surface of thick layer (*m* = 4, *n* = 3) (**b**) (see also Figure 1e,f). Curves 1 correspond to the multilayered film without gel; curves 2, film with pure gel (hydrogel) (*H_p_* = 0); curves 3 *H_p_* = 0.25 Oe; curves 4, *H_p_* = 0.5 Oe; curves 5, *H_p_* = 0.75 Oe. Other parameters used for calculations are the same as in Figure 2.

The influence of the stray fields induced by MNPs on the frequency dependence of the maximum MI ratio is illustrated in Figure 4. The presence of the stray fields results in a decrease of (Δ*Z*/*Z*)_max_ value with an increase of the concentration of MNPs in the ferrogel [7,34]. This fact is due to a decrease of the transverse permeability in the magnetic sub-layers under action of the stray fields. The dependences shown in Figure 4 describes qualitatively experimental results obtained previously [5,7,20,34]. The calculated frequency dependences of (Δ*Z*/*Z*)_max_ are similar for both the positions of the ferrogel layer, however, the maximum MI ratio is higher when the ferrogel is placed onto the surface of thin multilayer (Figure 4).

**Figure 5:**
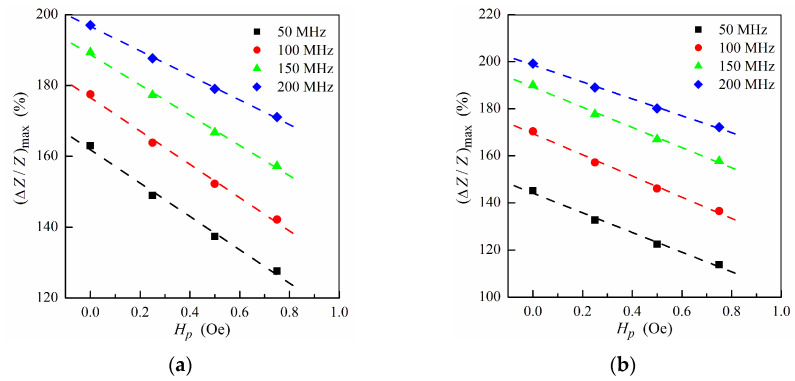
Maximum MI ratio (Δ*Z*/*Z*)_max_ as a function of the field *H_p_* at different frequencies for the multilayer with ferrogel placed onto the surface of thin layer (*m* = 3, *n* = 4) (**a**) and onto the surface of thick layer (*m* = 4, *n* = 3) (**b**) (see also Figure 1e,f). Other parameters used for calculations are the same as in Figure 2.

The MI response is sensitive to the stray field *H_p_* created by the MNPs, and hence the studied multilayer–ferrogel structure could be used to determine the concentration of the MNPs in the ferrogel in a simple and reliable way by MI measurements. In order to simulate the nanoparticle concentration dependence of the MI, the maximum MI ratio (Δ*Z*/*Z*)_max_ as a function of the stray fields intensity *H_p_* is obtained. Figure 5 shows the dependence of (Δ*Z*/*Z*)_max_ on *H_p_* calculated at different frequencies in a low and intermediate frequency range. For both ferrogel layer possible positions (at the surface of the thin or thick layer, see also Figure 1e,f), the dependence has a nearly-linear behavior for all frequencies, what can be useful for practical purposes of the rapid definition of the concentration of MNPs.

Let us introduce the concentration sensitivity of the MI response *S_c_*, which is defined as follows:

(14) $$S_{c}=-\frac{\partial {\left(\Delta Z/Z\right)}_{\max}}{\partial H_{p}}.$$

**Figure 6:**
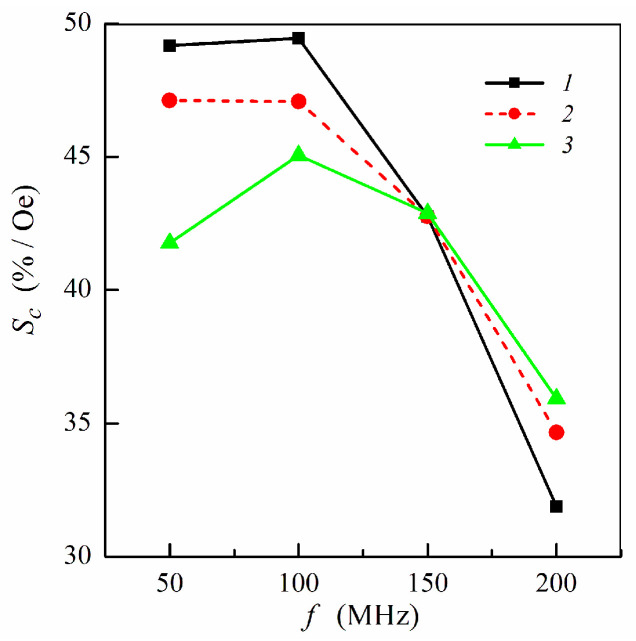
Frequency dependence of the concentration sensitivity *S_c_*: curve 1, symmetric multilayered film (*m* = *n* = 4); curve 2, non-symmetric multilayer with ferrogel placed onto the surface of thin layer (*m* = 3, *n* = 4); curve 3, non-symmetric multilayer with ferrogel placed onto the surface of thick layer (*m* = 4, *n* = 3). Other parameters used for calculations are the same as in Figure 2.

Figure 6 shows the frequency dependence of the concentration sensitivity calculated for different multilayer–ferrogel structures at selected frequencies. In a low-frequency range, maximum concentration sensitivity is achieved for symmetric multilayer. For the non-symmetric multilayers, the sensitivity *S_c_* is higher when the ferrogel is placed onto the surface of the thin magnetic layer of the MI element. For three studied multilayered films, the values of *S_c_* become the same at the frequency of 150 MHz. At higher frequencies, the concentration sensitivity drops and has higher values for the non-symmetric multilayers (Figure 6).

## 4. Discussion

Magnetic biosensors for MNPs detection in natural tissues are a recent imaging area. Although the possibility of the MNP detection inside living cells was demonstrated almost 15 years ago [13,14,15], the detection of the MNPs inside cells (typical sizes are 10–100 μm) is quite similar to an “in vitro” detection of biomolecular labels [1,11,39]. The principle of the magnetic label detection is an evaluation of the sum of the stray fields of all magnetizable particles. The MI effect showed its capability for sensing stray fields of MNPs at relatively large distances due to the very high sensitivity with respect to the applied magnetic field. The low sensitivity was a reason why for a long time the development of magnetic biosensors was no possible [39,40].

Recently we have reported w promising way for the detection of magnetic particles in blood vessels in the course of model experiments with multilayered [FeNi (100 nm)/Cu (3 nm)]_5_/Cu (500 nm)/[Cu (3 nm)/[FeNi (100 nm)]_5_ magnetoimpedance sensitive elements [41].

In addition, we discussed a hypothetical procedure in which the definition of the local concentration of magnetic carriers could be also used for correct definition of the start time for magnetic hyperthermia. Such an approach, i.e., combination of a number of functions assigned to the magnetic nanoparticles is a very hot topic, indicating some new theranostic directions [42].

Two asymmetric geometries for the detection of a flat ferrogel covering seem to be more useful for regenerative medicine cases. However, one can think about controlled drug release, biocompatible valves and actuators controlled by development of a magnetic field, or monitoring of the matrices for growing cells and tissues including growing conditions in a magnetic field as it was previously discussed for symmetric multilayered MI-sensitive elements [43]. In the case of controlled drug release it can be combined with magnetic hyperthermia [42,44] and control of the local concentration of MNPs by a magnetic field sensor.

As the next step, based on the model developed here, a module for commercial multi-physics software could be built. Our previous experience with symmetric MI structures in simpler cases was positive and fruitful [45]. Then, after verification, technology transfer to the high-tech industry should become possible.

The distance between the sensor surface and the magnetic label is a critically important parameter for the correct evaluation of the change in the output signal of a magnetic sensor. Ideal magnetic labels for biomedical detection are identical spherical superparamagnetic biocompatible MNPs, i.e., they all carry the same magnetic moments in an applied magnetic field of a certain strength. In our previous work, we proposed treating the assay of identical superparamagnetic labels as an additional layer of the multilayered structure [29]. Here, because of the theoretical comparison of symmetric and non-symmetric multilayered structures covered by a ferrogel layer we do observe the difference. The results obtained can be useful for improving the magnetoimpedance biodetector sensitivity.

In the described model, we were based on the results of our previous experiments, in which it was demonstrated that the magnetization of a polyacrylamide ferrogel with iron oxide nanoparticles increases linearly with increasing particle concentration [5,34]. Accordingly, the model assumes that the stray fields generated by the ferrogel are also proportional to the particle concentration. Moreover, the proportionality coefficient in the linear dependence of the field *H*_p_ on the concentration has not been discussed anywhere, since it cannot be determined from general considerations.

In principle, one can imagine a ferrogel in which the particles behave differently (for example, some sort of ordering of the antiferromagnetic type can occur). In this case, the dependence of the ferrogel magnetization on the concentration will indeed become nonlinear, but in any case, the magnetization will increase with increasing concentration. Qualitatively, the results of the model will not change for more complex magnetization behaviour, it is only necessary to change the linear dependence for another increasing function. However, the analysis of more complex dependences of the field *H*_p_ on the particle concentration is beyond the scope of this work.

The number of layers in MI structure analyzed here was proposed based on previous experimental and theoretical studies of FeNi-based multilayers [17,26,28]. High MI in a frequency range below 100 MHz is observed for [F/X]_m_/F/C/[F/X]_n_/F with m ≈ n and with the thickness of the [F/X]_m_ multilayered structures of about 0.5 μm. This roughly gives 20 < n, m < 3 for reasonable number of the sub-layers. A large m value results in the increase of the contribution of the interfaces, lower m causes transition into a “transcritical” state [30,31,46]–both causing the decay of MI effect value and sensitivity.

Special efforts for the development of MI biosensors is made both theoretical and experimental development of new designs of low field sensors with enhanced sensitivity, the possibility of magnetic noise reduction, simple design allowing the point-of-care usage, devises, adapted to microfluidic technologies, lab-on-a-chip packaging and sensor array design [47,48,49]. Magnetic biosensors are in their way of step-by-step replacement of many current diagnostic systems in laboratories and medical care points.

## 5. Conclusions

In this work, a theoretical description of the MI effect in non-symmetric multilayered elements covered by a ferrogel layer is developed. A model based on a solution of the Maxwell equations in the one-dimensional approximation allows one to find the distribution of electromagnetic fields in non-symmetric multilayers with ferrogel coverings. The symmetric distribution of the electromagnetic fields within the multilayer insures the highest values of the MI ratio. In the case of non-symmetric MI multilayered structures when the ferrogel is placed on the surface of the thin layer, the field distribution within the multilayer becomes more symmetric. When the ferrogel is placed onto the thick layer, the asymmetry in the field distribution is enhanced, resulting in the decrease of the MI ratio.

Within the low frequency range, placing the ferrogel onto the surface of thin layer increases the maximum MI ratio (Δ*Z*/*Z*)_max_. At frequencies above 150 MHz, the values of (Δ*Z*/*Z*)_max_ depend only slightly on the ferrogel position (onto thin or thick multilayer part). The presence of the stray fields results in a decrease of (Δ*Z*/*Z*)_max_ value with an increase of the concentration of MNPs in the ferrogel due to a decrease of the transverse permeability in the magnetic sub-layers under action of the stray fields. The calculated frequency dependences of (Δ*Z*/*Z*)_max_ are similar for both the positions of the ferrogel layer. However, the maximum MI ratio is higher when the ferrogel is placed onto the surface of a thin layer. The studied multilayer–ferrogel structure could be used to determine the concentration of the MNPs in the ferrogel by MI measurements, since the MI response is sensitive to the stray field *H_p_*. For both ferrogel layer positions (onto the surface of thin or thick layer), the concentration dependence (Δ*Z*/*Z*)_max_(*H_p_*) has a nearly linear behavior for all frequencies. The sensitivity of the MI response for non-symmetric multilayers calculated for different multilayer–ferrogel structures at selected frequencies is higher when the ferrogel is placed onto the surface of a thin magnetic layer.

## Data Availability

Data available from the corresponding author upon reasonable request.
